# Exosomes in Cardiovascular Medicine

**DOI:** 10.1007/s40119-017-0091-9

**Published:** 2017-05-19

**Authors:** Iain M. Dykes

**Affiliations:** School of Clinical Sciences, University of Bristol, Bristol, United Kingdom

**Keywords:** Acute myocardial infarction, Biomarker, Cardiovascular disease, Circulating micro-RNA, Drug delivery, Exosome, Micro RNA, Nanoparticle, Stem cell therapy, Troponin

## Abstract

Exosomes are small, extracellular membrane-bound particles that mediate intercellular transport of a cytosolic cargo. Exosomal transfer of micro-RNA can modify gene expression in targeted cells. Exosome-based endocrine/paracrine signaling has been shown to be involved in a wide range of physiological processes including those associated with cardiovascular injury and disease, but remains relatively poorly understood. Exosomes offer great potential to the clinical field, with applications in both diagnostics and therapeutics. A stable, circulating form of micro-RNA exists in blood protected from endogenous nucleases. This population of micro-RNA, which includes both exosomal and non-exosomal fractions, may be isolated from blood and exploited as a novel disease biomarker with the potential to deliver increased specificity and rapid diagnosis compared to conventional biomarkers. Exosomes also offer a natural drug-delivery vehicle, providing immune evasion and specific targeting through engineering of surface-displayed ligands. Much of the cardioprotective and regenerative benefits of stem-cell grafts are now thought to derive from paracrine signaling rather than direct tissue incorporation and therefore stem cell-derived exosomes offer the potential for a convenient cell-free therapeutic option, eliminating many of the risks and variability associated with stem-cell therapy. In this review, we consider the potential applications of this emerging field to cardiovascular medicine, taking myocardial infarction as our primary example.

## Exosomes Represent a New Paradigm for Endocrine/Paracrine Signaling

Exosomes are small membrane-bound particles actively released by cells under both physiological and stressed conditions. Exosomes carry a cargo of RNA and soluble proteins and display specific receptors on their surface that may function in targeting to specific receiver cells. RNA carried by exosomes includes both mRNA and micro-RNA (miR) and these RNAs are able to modify gene expression within receiver cells [1–3]. Exosomes have been shown to mediate a number of paracrine signals within the cardiovascular system, for example, between the vascular endothelium and smooth muscle [4], between cardiac fibroblasts and cardiomyocytes [5], and between vascular smooth muscle cells [6]. Exosomes of cardiovascular origin are also present in the pericardial fluid [3] and in blood [7, 8], indicating their likely involvement in endocrine signaling. Changes in circulating exosomes are linked to diseased states [7–9], suggesting their potential as a diagnostic biomarker. Exosomes can be isolated from the conditioned media of cells in vitro and this, together with the relative ease of engineering both exosome cargo and surface displayed proteins offers much potential for therapeutic drug delivery.

Exosomes represent one of three broad classes of extracellular vesicle. Membrane blebbing from cells undergoing apoptosis generates so-called apoptotic-bodies [10, 11] of a size generally between 0.5 and 2 µm. Although apoptotic bodies have been shown to mediate cell signaling under certain circumstances [12, 13], they are normally regarded as a passive by-product of cellular breakdown and thus of limited interest. Ectosomes (also known as shedding microvesicles) are produced by outward-budding (ectocytosis) of the plasma membrane, and are generally larger than exosomes [14, 15]. In contrast, exosomes are actively produced in the endosomal pathway. Exosome biogenesis involves endocytosis from the plasma membrane followed by subsequent invaginations from the endosomal membrane to create a multivesicular body [16]. Upon fusion of the outer membrane with the plasma membrane, the intraluminal vesicles are released as exosomes into the extracellular space. This review will be focused specifically on exosomes, although it should be noted that most biological samples such as blood contain a mixture of ectosomes and exosomes, which may be difficult to separate [17, 18]. However, there are many similarities between these two particles, both being fundamentally dependent on budding from the plasma membrane, and they may share a number of biological functions [15].

Exosomes consist of a lipid bilayer and a cytosol, within which is transported a “cargo” of soluble molecules. Exosome membrane proteins include those involved in membrane budding and fusion (common to all exosomes, as well as to lysosomes, which share a common endosomal origin) as well as cell-specific receptors believed to be involved in exosome targeting to receiver cells [16]. The cargo includes proteins, mRNA, and both large and small noncoding RNA. Different mechanisms are used to load protein and RNA cargoes. Protein loading mechanisms are the better understood and include the endosomal sorting complexes required for transport proteins (ESCRT) and tetraspanins [19]. RNA loading mechanisms are less clear, but some studies have identified sequence motifs enriched in exosomal miRs that are required for their interaction with RNA binding proteins [19, 20]. The exosome membrane serves to protect the cargo from nucleases present in the blood as well as from the immune system, exosomes are thus able to deliver a complex signal consisting of multiple proteins and RNAs to the receiver cell, which they do through an active uptake mechanism either by fusing with the plasma membrane or by endocytic internalization followed by fusion with the endosome membrane. This can be visualized with lipophilic dyes [21].

## Exosomes and Circulating miR as Biomarkers for Cardiovascular Disease

Biomarkers are a useful diagnostic tool in many areas of cardiovascular medicine. We will here take acute myocardial infarction (AMI) as an example. ST segment elevation myocardial infarction (STEMI), the most severe form of the disease in which a coronary artery is completely blocked, is treated by reperfusion, and this is most effective if performed soon after the infarction [22, 23]. Any delay in the time to reperfusion following a patient’s arrival at the hospital is associated with a higher risk of in-hospital mortality and current guidelines recommend treatment within 90 min [22, 23]. It is therefore critical to rapidly confirm a suspected STEMI. This is currently performed by analysis of cardiac troponin (TNNI3, TNNT2) plasma protein levels. These proteins are highly expressed in cardiomyocytes and are released into circulation upon infarction-induced necrosis [24]. However, troponin levels do not begin to rise until 4–6 h after infarction [24] and thus reperfusion must be initiated before these results are obtained [22]. Furthermore, any condition leading to such necrosis, such as sepsis or cardiotoxicity following chemotherapy, can give a false-positive result [24]. There is therefore a need for a biomarker that is both more specific for AMI and that can provide a faster diagnosis.

Both earlier diagnosis and increased specificity could be achieved by using a “pre-necrotic” biomarker, a signal actively released by the injured heart before necrosis begins. Hypoxia has been shown in vitro to induce cardiomyocyte release of exosomes carrying a protein cargo consisting of the cytokine TNFα [25] and the heat shock protein HSP60 [26]. Therefore, it is not unreasonable to suggest that in vivo a cardiomyocyte that is in a state of hypoxia following AMI, but is not yet necrotic, might release exosomal endocrine signals into the blood. In support of this hypothesis, we know that there is an increase in plasma exosomes in the 2 days following coronary artery bypass surgery [7], indicating that injury to the cardiovascular system leads to exosome release.

Since the discovery that micro RNA exists in a stable form in the blood [27], much interest has focused on the potential of so-called circulating microRNA as a biomarker, and evidence is accumulating that these may be useful in the diagnosis of diseases such as cancer [27] as well as cardiovascular disease [28, 29]. Stabilized miR seems to be present in blood in two forms: it may be protected from nucleases either by an exosome-based mechanism or by extra-vesicular complexing to proteins such as the silencing effector protein AGO2 [30] or the RNA binding protein Nucleophosmin-1 [31]. Some of this protein-bound miR may be released upon necrosis following AMI, although it should be noted that there is evidence both for active release of protein-complexed miR [31] and for active sorting such that particular miRs are bound to proteins while others are loaded into vesicles [30]. In addition, AGO2 has been shown to be present in exosomes [3], suggesting that some protein-bound miR may be of exosomal origin.

Most studies of AMI biomarkers have taken a candidate gene approach, tending to focus on miRs known to be highly expressed in the heart. For example, the cardiomyocyte-specific miR-208a [32, 33], which is expressed from an intron within alpha myosin heavy chain [34], and the more general muscle-expressed miRs such as miR-1-1/miR1-2, miR-133a/b, and miR-499 [32, 33, 35, 36] have been shown to increase following AMI by a number of groups. miR-1-2 and miR-133a are produced from the same primary transcript—a long noncoding RNA called *MIR133A1HG*—and share a common promoter [37] while miR-499 is expressed from an intron within another myosin heavy chain gene, *MYH7B*, expressed in cardiomyocytes as well as in skeletal and smooth muscle [38]. One problem with looking at miRs highly expressed in the heart is that one might argue that their presence in circulation results from passive release following necrosis, and indeed some do correlate with cardiac troponin [35, 39]. However, there is also evidence that some rise more quickly [39]. An analysis of blood taken from patients undergoing transcoronary ablation of septal hypertrophy (TASH)—a surgical intervention resembling AMI, in which overgrowth of the ventricular septum is reduced by inducing a controlled infarction of the affected area—which allows blood sampling at early timepoints, found significant plasma increases in miR-1 and miR-133a only 15 min after surgery [33]. Thus, there is circumstantial evidence for a pre-necrotic release mechanism.

In vitro data indicate that cells select specific miRs for release while retaining others [40] and that not all miRs found in exosomes are expressed by the parent cell [1]. Injury induces gene expression changes, for example hypoxia induces expression of miR-210 in cardiomyocytes [41]. Thus, it is perhaps not logical to assume that miRs expressed by healthy cardiomyocytes will be those released upon injury. Indeed, unbiased biomarker screens utilizing techniques such as microarray analysis have identified many unexpected changes. Within the heart itself, one study found 47 changed miRs 7 days post AMI [42]. Changes in circulating micro RNA may be even higher: one study found 121 significantly changed miRs following AMI (of which 63% are downregulated) [43], while another found 20 upregulated and 14 downregulated miRs [35]. Many of these dysregulated circulating miRs are not cardiomyocyte-specific, but as the authors note [43], there is no particular reason to assume that changed miRs should be of cardiomyocyte origin, other cell types such as endothelium may also contribute to the AMI response.

Despite these data showing that gene expression changes upon injury, indirect evidence does support the hypothesis that muscle-type miRs highly expressed under normal conditions may be released upon injury. Work in a mouse model of AMI shows that miR-133a is downregulated in cardiomyocytes within the infarct region [44]. This coincides with the time that, based on analysis of patient serum, we assume that miR-133 exosomes are produced and thus suggests that miR-133a present in healthy cells may be targeted to exosomes upon injury. Direct evidence for this is lacking at present, but these results are intriguing, not least because the same authors demonstrated that miR133a-containing exosomes released by H9c2 rat embryonic myoblasts are functional, as shown by reduced expression of a luciferase reporter when applied to HEK293 cells [44].

**Fig. 1:**
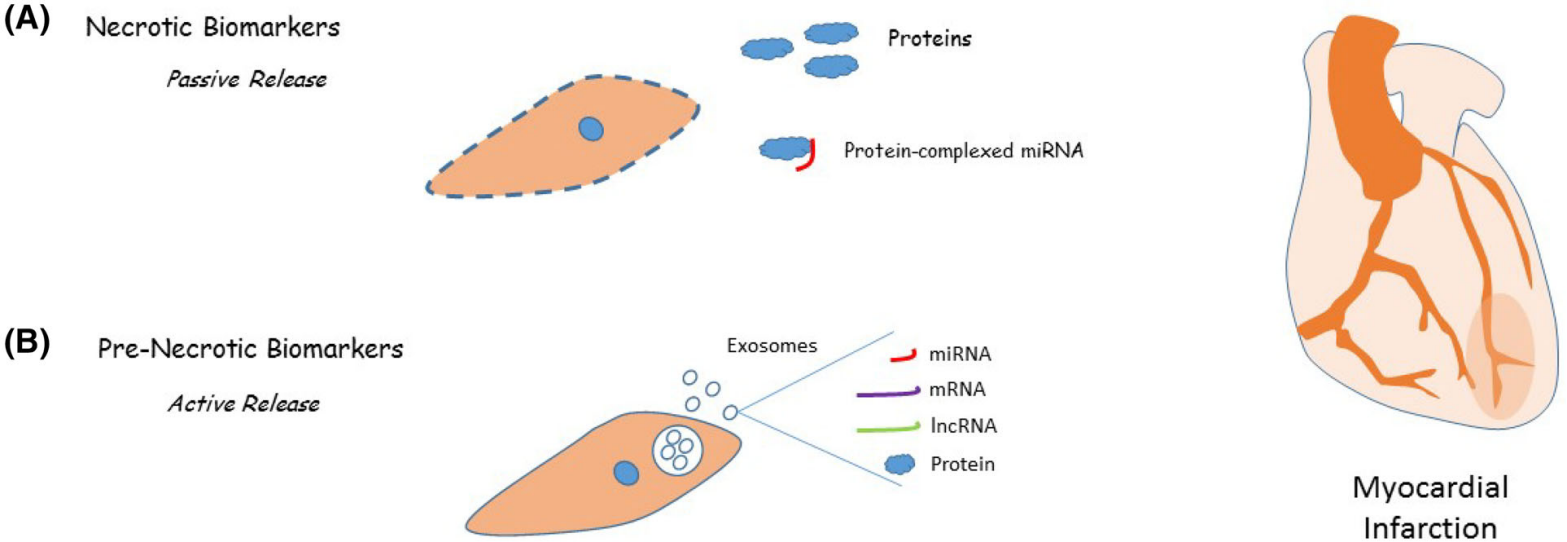
Diagnostic exosome applications. **a** Current diagnosis of acute myocardial infarction relies on detection of proteins such as cardiac troponin, which are passively released into the circulation upon cardiomyocyte necrosis. Necrosis may also release protein-complexed miRNA into circulation. **b** Active release of exosomes by injured cells offers the potential for a faster and more specific pre-necrotic diagnosis. Exosomes may be released within minutes of infarction and carry a complex cargo, which may include miRNA, mRNA, lncRNA, and proteins, offering potential for detection of a unique infarction molecular fingerprint

Exosomes carry a complex message consisting of a mix of several molecules. Therefore, the true value of exosomes as biomarkers may be to define a molecular signature for AMI (Fig. 1). It is likely that this will depend not on analysis of one or two markers as in the studies above, but on a more sophisticated analysis of combinations of co-released miRs, and perhaps other cargo such as proteins (exosomal clusterin is increased in pericardial fluid following AMI [45]) or lncRNA [46, 47]. Towards this goal, a recent paper has proposed that a panel of differentially expressed miRs can be used to differentiate Takotsubo cardiomyopathy from AMI [48]. Takotsubo cardiomyopathy has similar symptoms to AMI but is caused by stress-induced changes in ventricular function rather than by coronary artery occlusion. The study found that expression analysis of synchronised changes in four circulating miRs could differentiate the two conditions.

The use of circulating miRs as biomarkers is gaining increasing interest within many areas of cardiovascular medicine beyond AMI including coronary artery disease, atrial fibrillation, hypertrophic cardiomyopathy, and heart failure [49–51]. For example, there is evidence that the blood levels of miRs normally expressed in the endothelium are decreased in patients with coronary artery disease [52]. Valve disease is another area in which circulating miR biomarkers have been explored [53]. A circulating miR signature for the congenital disease bicuspid aortic valve (BAV) consisting of miR-122, miR-130a, and miR-486 has been proposed [54] and this seems to differ from the profile of miR expression changes observed within the valve tissue itself [55].

Although circulating miRs and exosomes in general certainly show potential as a biomarker, a number of technical challenges remain to be overcome before they can be routinely utilized in the clinic [56, 57]. Assaying miR by RT-qPCR is a more complex and time-consuming procedure than a simple ELISA assay and this may negate some of the temporal advantage over the troponin assay. In addition, low miR expression levels make accurate quantification difficult, endogenous controls are not standardized, and a lack of reproducibility has also been noted in many such studies.

## Exosomes Offer an Alternative to Stem Cell Therapy

Ischemia resulting from myocardial infarction results in extensive cardiomyocyte necrosis, formation of a fibrotic scar, and compensatory ventricular remodeling, often leading to heart failure at a later date [58]. Surgical intervention such as coronary artery bypass graft can restore blood flow and limit damage but cannot regenerate tissue already lost. Stem cell therapy offers hope in this regard [59, 60]. Stem cells derived from a variety of sources including bone marrow hematopoietic (HSCs) [61] and mesenchymal (MSCs) [62] stem cells as well as embryonic stem cells (ESCs) [63] have been shown to differentiate into cardiomyocytes in animal models or to improve outcomes in clinical trials.

The adult heart has been shown to contain a small population of endogenous stem cell-like cells, marked by expression of c-kit [64] and these also offer hope for regeneration. Resident stem cells may be isolated and grown from myocardial biopsies as a result of their capacity to form multicellular clusters known as cardiospheres and these cardiosphere-derived cells (CDCs) may be used for therapy [65]. Although transplant of autologous CDCs derived from the patient themselves would be ideal, and these have been shown to reduce scarring and improve cardiac function in a phase one clinical trial [66], this procedure is limited by time-consuming isolation and growth protocols. Allogenic cell transplants, in which the cells are not derived from the patient, offer a more standardized, off-the-shelf solution.

Surprisingly, allogenic intramyocardial injection of CDCs into the infarct periphery in rats does not result in a large immune response [67]. This seems to be because the cells themselves do not survive long in the host (less than 1% remain after 3 weeks) [67], and their main function seems to be a paracrine one. Indeed, pre-treatment of human CDCs with a drug to inhibit exosome secretion eliminates their beneficial effects when injected into mice following an induced MI [68]. In fact, evidence is accumulating suggesting that survival of engrafted stem cells is often poor [69] and that the beneficial effects of many stem cell therapies, including embryonic [70], induced pluripotent [71], ESC-derived cardiac progenitors [72, 73], and mesenchymal [74, 75] result not because the injected stem cells differentiate into new tissue but instead because they seem to act as a paracrine source of exosomal growth factors. For example, in a mouse model of AMI, intramyocardial injection of exosomes derived from ESCs increased the number of resident progenitor cells as well as increasing capillary density around the infarct, resulting in improved cardiac function [70]. Exosomes derived from cardiac progenitor cells isolated from the atrial appendages of neonatal children improved heart function in a rat AMI model [76]. Similarly, MSC exosomes have been shown to reduce infarct size in a mouse model [74] while pre-treatment of CDCs with MSC-derived exosomes improved survival and reduced fibrosis in a rat AMI model [77]. Exosomes have been shown to exert their regenerative effects via a number of mechanisms including stimulating differentiation of endogenous resident stem cell populations [70], by conferring a cardioprotective effect on existing cardiomyocytes [78, 79] and by promoting angiogenesis [80].

Exosomes have also been shown to have a beneficial effect in other cardiovascular settings. For example, MSC exosomes have been shown to reduce vascular remodeling in a mouse model of pulmonary hypertension [81], exosomes derived from CD34+ HSCs have been shown to mediate the angiogenic properties of these cells in subcutaneous or corneal mouse implants [80], while endothelial progenitor cell (EPC) exosomes mediate neovascularization in a mouse model of hind limb ischemia [82]. In vitro studies have demonstrated that EPC exosomes bind to and are taken up by endothelial cells of the vasculature and are able to transfer mRNA to these cells [83]. Cardiac fibroblasts have been shown to produce exosomes, which transfer miRs to receiver cells, and this is important in cardiac hypertrophy [5].

An interesting addition to these studies is the finding that exosome-mediated communication from cardiac endothelial cells can improve survival of engrafted cardiac progenitor cells (CPCs). Co-injection of a DNA construct encoding the hypoxic-induced transcription factor HIF1 improved survival of CPCs in a mouse AMI model [84]. This seems to be because endogenous endothelial cells expressing HIF1 driven by the vector produce exosomes with a higher concentration of the pro-survival miRs miR-126 and miR-210, which are then taken up by CPCs and induce a metabolic switch in these cells [84].

**Fig. 2:**
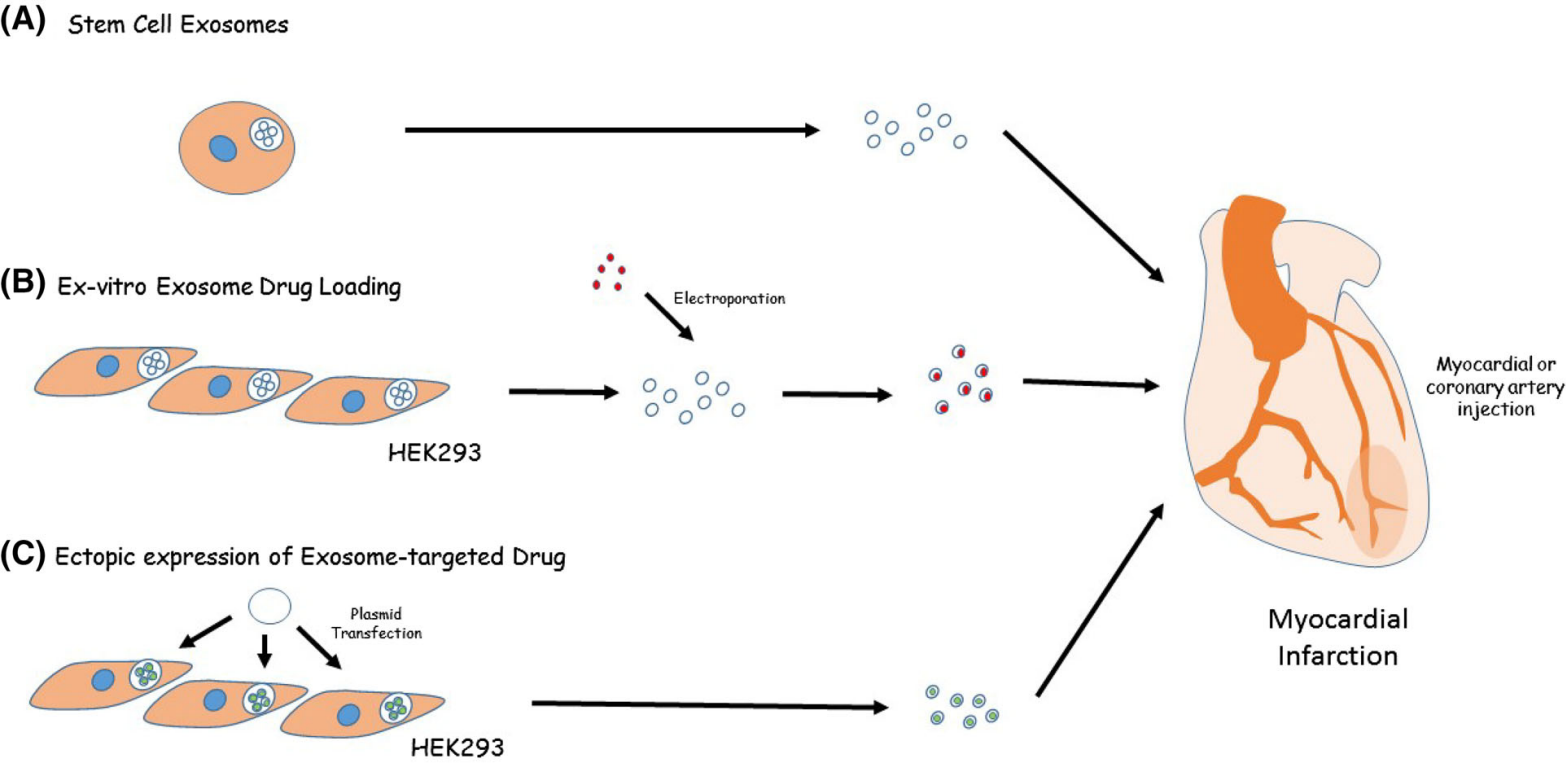
Therapeutic exosome applications. Exosomes have shown much promise in the treatment of myocardial infarction. **a** Much of the therapeutic benefits of stem cells appear to be mediated by paracrine exosome-based mechanisms, and thus extraction and delivery of such exosomes offers a lot of potential for a form of cell-free stem cell therapy, reducing the risks of stem cell injection and allowing production of a standardized off-the-shelf product. **b** Exosomes may be engineered for use as a natural drug delivery vehicle. Exosomes released from cells grown in vitro may be loaded with RNA by electroporation. Lipophilic drugs such as doxorubicin may be directly loaded into exosomes. **c** An alternative strategy is to transfect cells with an expression plasmid encoding an exosome-targeted protein, mRNA, or miRNA to take advantage of the cell endogenous sorting mechanisms to achieve exosomes-loading

The therapeutic potential of these observations are enormous. If, as seems to be the case, exosome-mediated signals produced by stem cells are more important to regeneration than the cells themselves, it may be possible to isolate this factor to produce a “cell-free” form of stem cell therapy (Fig. 2). This has the advantage of convenience, offering the potential for an “off-the-shelf” solution, and also the potential for bulk production in vitro. Finally, exosomes therapies may reduce the risks associated with stem cell therapies.

## Engineering of Exosomes for Specific Targeting and Drug Delivery

Synthetic vesicles (liposomes) have been used as an artificial drug delivery vehicle for over 40 years [85]. These vesicles consist of a simple lipid bilayer, sometimes conjugated to molecules such as polyethylene glycol to aide immune evasion. Exosomes offer the potential for a natural drug delivery system, and have a number of advantages over liposomes. Exosomes, in contrast to liposomes, express a complex array of proteins within the membrane and these may be modified to display surface markers. Exosome targeting to a specific receiver cell or tissue is generally achieved by surface display of a specific ligand. Use of a short peptide is preferable to use of a whole protein because this is less likely to stimulate an immune response, and is easier to clone [86]. This is most easily achieved by transfecting cells in vitro with an expression construct encoding a fusion protein containing the ligand of interest fused to an endogenous exosome membrane protein [86, 87]. Examples of suitable exosome proteins include MFGE8 and LAMP2B.

MFGE8 (Lactadherin) is a secreted glycoprotein first isolated from milk, but later shown to be present in the exosomal membrane of dendritic cells [88]. It contains two motifs, an RGD domain, which interacts with integrins, and a C1C2 domain, which mediates its interaction with membranes, including those of exosomes. Of interest to cardiovascular medicine is the observation that MFGE8 is expressed by blood vessels and blocking antibodies raised against it inhibited VEGF-mediated neovascularization in a rodent limb ischemia model [89]. This is thought to involve interaction of the RGD domain with integrins [89], although the possibility that MFGE8 could be presented to target cells on exosomes was not addressed. Expression of ectopic proteins fused to the MFGE8 C1C2 domain (without the integrin-interacting RDG domain) allows targeting to the exosome membrane [90], although it should be noted that MFGE8 is not exclusively expressed in exosomes but is also present in other species of extracellular vesicle [91]. Lysosome-associated membrane proteins (LAMPs) are transmembrane glycoproteins that are specifically targeted to the lysosome membrane by a motif at the C-terminal [92]. Fusion of a peptide from a rabies viral glycoprotein (which binds the acetylcholine receptor) to LAMP2b successfully targeted exosomes to the mouse brain [87, 93].

Exosomes may also be modified to carry an exogenous cargo. Cargo-loading strategies can be divided into those that employ direct loading of isolated exosomes and those that take advantage of endogenous cellular cargo loading mechanisms [85, 94]. The former is often used for RNA and siRNA loaded directly into purified exosomes by electroporation is functional [87, 93], despite the fact that this method seems to precipitate RNA [95]. Isolated exosomes can also be loaded with a number of lipophilic molecules including anticancer drugs such as doxorubicin [94]. Transfection of donor cells with an expression plasmid can be sufficient to generate RNA-containing exosomes and this strategy has been used to produce miR-146b exosomes [96]. This method has also been used to generate exosomes carrying a cargo of synthetic anti-miR [97] indicating an alternative therapeutic strategy. An alternative approach is to manipulate cell-sorting mechanisms by expressing a fusion protein consisting of an exosomal membrane protein (LAMP2B) linked to an RNA-binding domain (bacteriophage MS2) such that over-expression of an RNA carrying the motif recognized by MS2 results in production of RNA-containing exosomes [98]. This method is perhaps best suited to mRNA rather than short RNAs. This article is based on previously conducted studies and does not involve any new studies of human or animal subjects performed by any of the authors.

## Conclusions

Exosomes show great potential to improve both diagnosis and therapy within cardiovascular medicine, but many questions remain and we are some way away from bringing them into the clinic.

Pre-clinical studies have demonstrated that exosome-based signaling is both widespread and important in the cardiovascular system and the evidence suggests that exosomes are released into circulation, yet we know very little of the function of this novel form of endocrine signaling. While exosome-based biomarkers offer great potential in the development of unique signatures for specific cardiovascular disease, a greater understanding of the biological functions of these particles is needed before we can draw firm conclusions based on their blood levels. For example, many studies of AMI biomarkers make the assumption that the same miRs expressed in healthy cardiomyocytes will be released upon injury, but why should this be the case? Perhaps novel miR signaling pathways are activated upon injury, and functional work on the many novel miR biomarkers identified in microarray screens [35, 43] should be a fruitful avenue for exploration. Additionally, it is difficult at present to distinguish exosomal miR from other forms of circulating miR, as well as to distinguish exosomes from other classes of nanoparticles, and methods to improve these distinctions are likely to be valuable in the development of biomarkers. Inconsistencies within the current data are attributable in part to biased, non-random selection of potential biomarkers [51] as well as to technical challenges associated with these assays [56, 57]. Of particular importance to their future utility will be the development of more sensitive assays for quantification of the low levels of miR found in the blood.

Similarly, in therapeutics, exosomes offer great potential as a result of their ability to evade the immune system and to offer specific targeting. Yet our knowledge of exosome synthesis and cargo-loading pathways are limited, and as our knowledge of the basic science increases, this should improve our ability to manipulate exosomes for therapeutic use. In particular, we do not yet fully understand how RNA is sorted and loaded into exosomes, while many proteins used to target ligands to exosome membranes are also expressed in lysosome membranes, and thus are not specific to exosomes.

Despite these challenges, the emerging field of exosome signaling offers many opportunities for cardiovascular medicine.
