# Correction to: Exosomes in Cardiovascular Medicine

**DOI:** 10.1007/s40119-018-0105-2

**Published:** 2018-03-08

**Authors:** Iain M. Dykes

**Affiliations:** School of Clinical Sciences, University of Bristol, Bristol, UK

## Correction to: Cardiol Ther (2017) 6:225–237 10.1007/s40119-017-0091-9

This article was originally published under a Creative Commons Attribution-NonCommercial 4.0 International License (CC BY-NC 4.0), but has now been made available under a Creative Commons Attribution 4.0 International License (http://creativecommons.org/licenses/by/4.0/), which permits unrestricted use, distribution, and reproduction in any medium, provided you give appropriate credit to the original author(s) and the source, provide a link to the Creative Commons license, and indicate if changes were made. The PDF and HTML versions of the paper have been modified accordingly.

